# Supplementary material for: Analysis of context-specific KRAS–effector (sub)complexes in Caco-2 cells
Source: Life Sci Alliance. 2023 Mar 9;6(5):e202201670. doi: 10.26508/lsa.202201670 (PMC9998658; doi:10.26508/lsa.202201670)

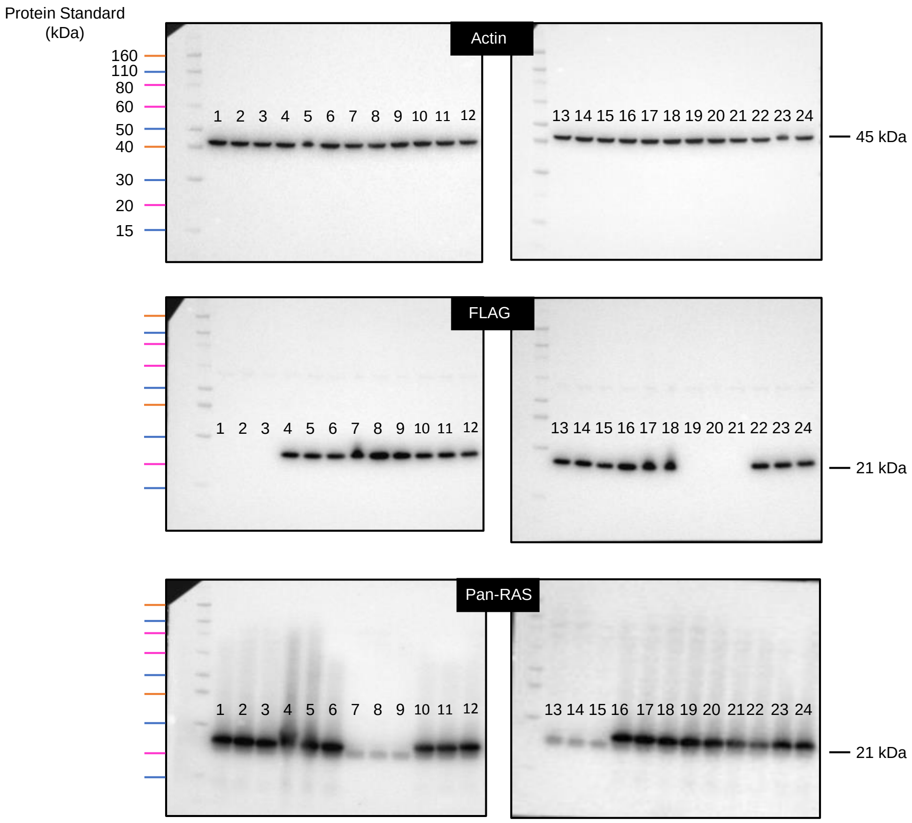

__Full blots:__ *Western blot confirmation of exogenous expression of FLAG-tagged KRAS (either WT, G12D, G12V or G12C) in Caco-2 cells using an anti-FLAG antibody and an anti-Pan-RAS antibody. β-actin was used as a loading control. Each antibody is shown on a separated and individual full blot and the data are shown in N=3 independent experiments and analysed with ImageJ. The protein standard used is the Novex Sharp Pre-Stained Protein Standard (ThermoFisher). 1:NT_01, 2:NT_02, 3:NT_03, 4:KRAS_WT_01, 5:KRAS_WT_02, 6:KRAS_WT_03, 7:KRAS_G12D_01, 8:KRAS_G12D_02, 9:KRAS_G12D_03, 10:KRAS_G12V_01, 11:KRAS_G12V_02, 12:KRAS_G12V_03, 13:KRAS_G12D_01, 14:KRAS_G12D_02, 15:KRAS_G12D_03, 16:KRAS_G12C_01, 17:KRAS_G12C_02, 18:KRAS_G12C_03, 19:NT_01, 20:NT_02, 21:NT_03, 22:KRAS_WT_01, 23:KRAS_WT_02, 24:KRAS_WT_03.*

Blot shown in manuscript

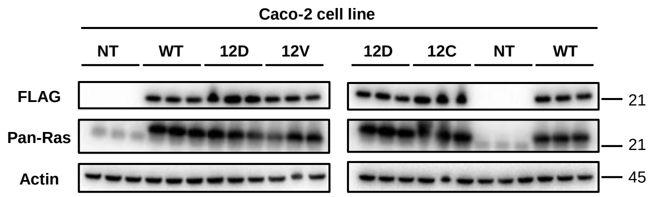

Supplement: Supplementary file 1 [file LSA-2022-01670_SdataFS2.pdf]
