# Supplementary material for: Analysis of context-specific KRAS–effector (sub)complexes in Caco-2 cells
Source: Life Sci Alliance. 2023 Mar 9;6(5):e202201670. doi: 10.26508/lsa.202201670 (PMC9998658; doi:10.26508/lsa.202201670)

Western Blot RAS expression after transfection in different culture contexts

Blot 1

Pan-RAS

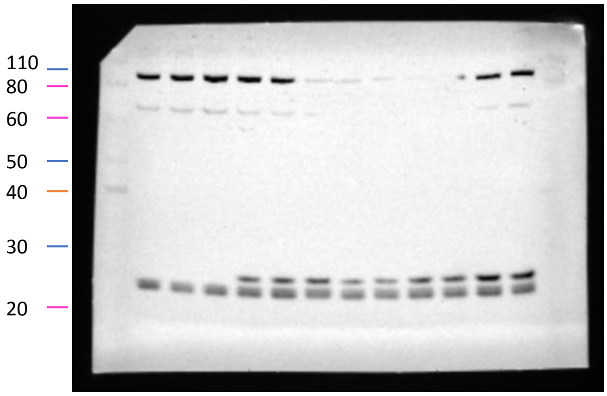

Blot 2

Pan-RAS

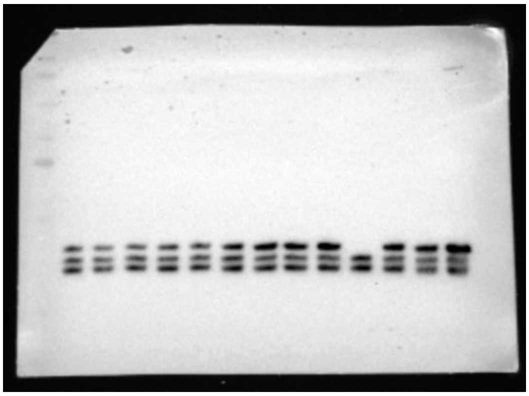

Actin-β (Blot 1)

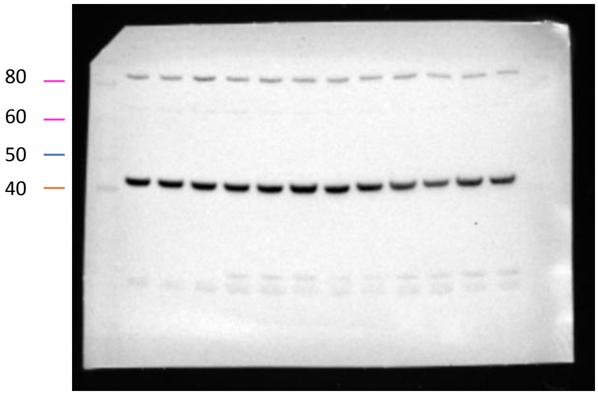

Actin-β (Blot 2)

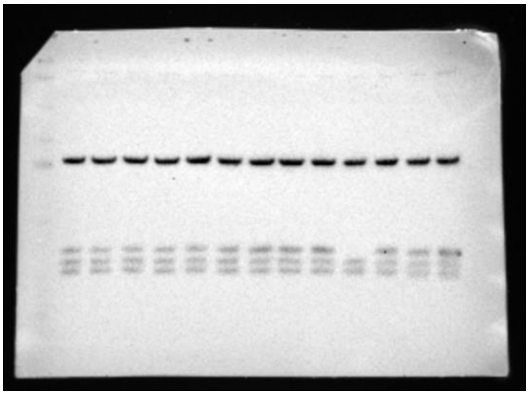

Blot shown in Fig. S3

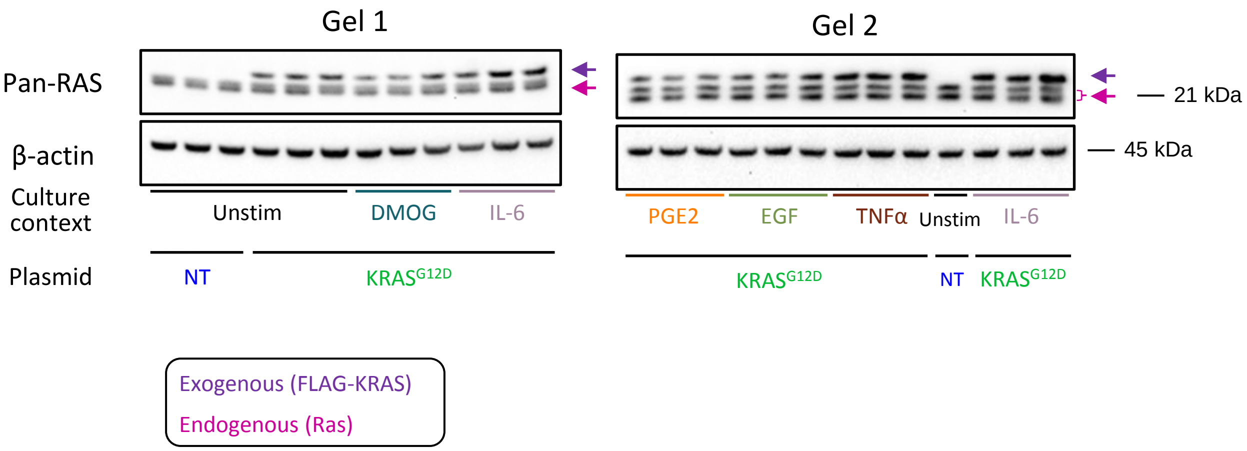

Supplement: Supplementary file 2 [file LSA-2022-01670_SdataFS3.pdf]
