# Supplementary figures and images for: Analysis of context-specific KRAS–effector (sub)complexes in Caco-2 cells
Source: Life Sci Alliance. 2023 Mar 9;6(5):e202201670. doi: 10.26508/lsa.202201670 (PMC9998658; doi:10.26508/lsa.202201670)

# Western Blot FLAG expression evolution until 72h post transfection

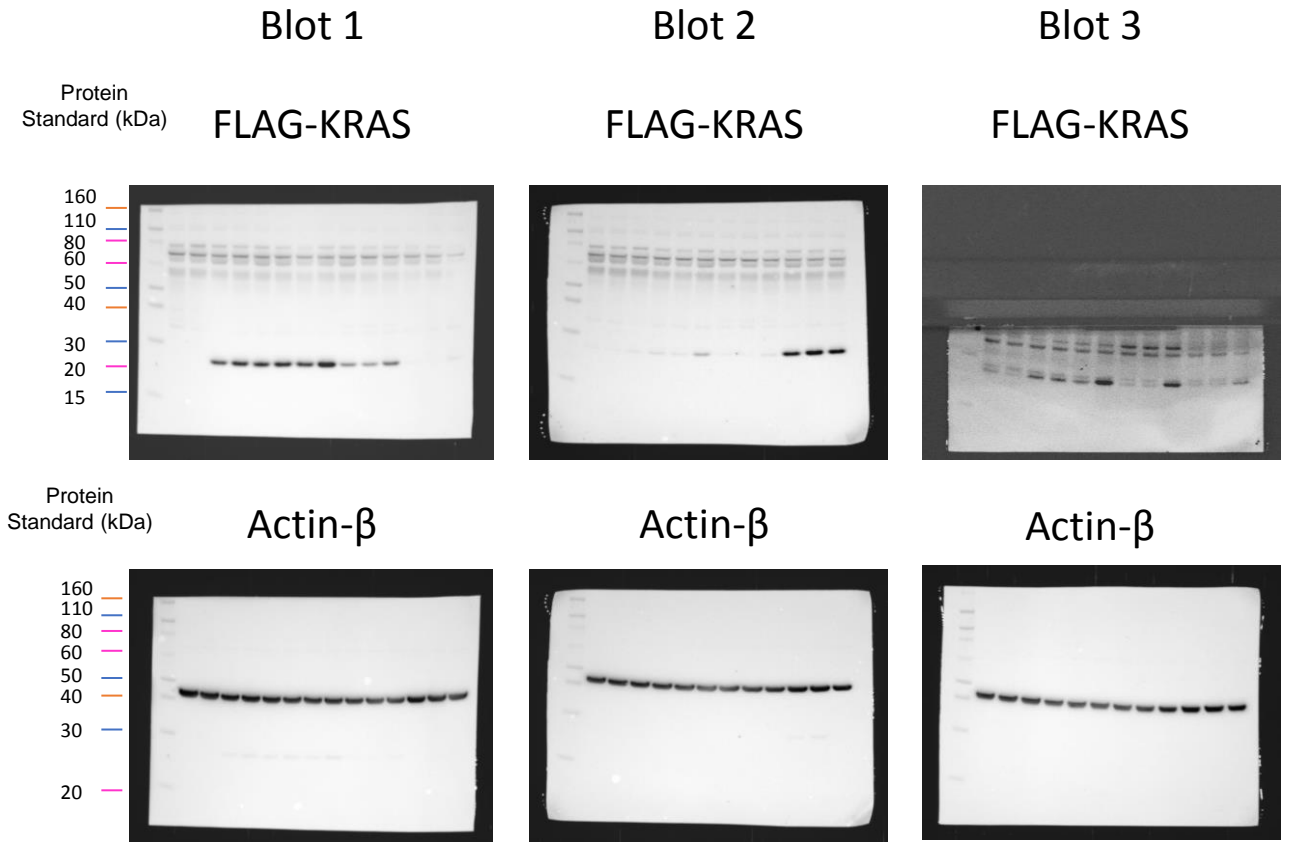

Blot shown in Fig. S13

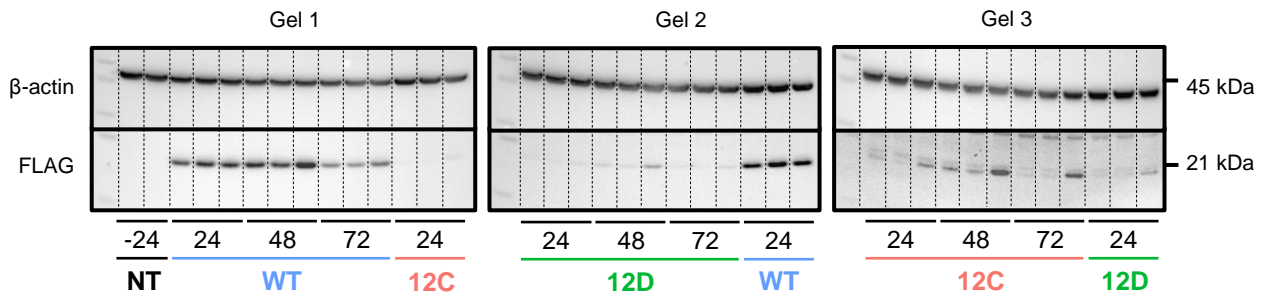

Supplement: Supplementary file 3 [file LSA-2022-01670_SdataFS13.pdf]
