# Supplementary material for: Analysis of context-specific KRAS–effector (sub)complexes in Caco-2 cells
Source: Life Sci Alliance. 2023 Mar 9;6(5):e202201670. doi: 10.26508/lsa.202201670 (PMC9998658; doi:10.26508/lsa.202201670)
Supplement: Supplementary file 4 [file LSA-2022-01670_TableS1.docx]

| **GO term identification** | | **Pairwise comparison** | | | **Statistics** | |
| --- | --- | --- | --- | --- | --- | --- |
| **ID** | **Term** | **Context effect** | **Group higher** | **Group lower** | **LS mean difference** | **p-adjusted** |
| GO: 0050673 | Epithelial cell proliferation | Culture | DMOG | IL-6 | 2.227 | 4.36E-09 |
|  |  |  | DMOG | Unstim | 2.891 | 4.08E-09 |
| GO: 0050679 | Positive regulation of cell population proliferation | Culture | DMOG | IL-6 | 2.682 | 3.46E-07 |
|  |  |  | DMOG | Unstim | 5.347 | 4.07E-09 |
|  |  |  | IL-6 | Unstim | 2.665 | 2.66E-04 |
| GO: 0006006 | Glucose metabolic process | Culture | DMOG | IL-6 | 1.116 | 3.16E-04 |
|  |  |  | DMOG | Unstim | 2.738 | 4.07E-09 |
|  |  |  | IL-6 | Unstim | 1.622 | 1.54E-05 |
| GO: 0010906 | Regulation of glucose metabolic process | Culture | DMOG | IL-6 | 0.781 | 4.14E-04 |
|  |  |  | DMOG | Unstim | 2.905 | 4.07E-09 |
|  |  |  | IL-6 | Unstim | 2.124 | 4.07E-09 |
|  |  | Genetic | G12C | WT | 0.846 | 1.18E-06 |
|  |  |  | G12D | WT | 0.763 | 1.50E-05 |
|  |  | Genetic:Culture | G12C:DMOG | WT:DMOG | 1.603 | 6.28E-03 |
|  |  |  | G12D:DMOG | WT:DMOG | 1.513 | 1.53E-02 |
|  |  |  | G12C:IL-6 | WT:IL-6 | 1.365 | 3.30E-02 |
|  |  |  | G12D:DMOG | G12D:Unstim | 3.386 | 4.59E-09 |
|  |  |  | G12D:IL-6 | G12D:Unstim | 2.839 | 4.45E-07 |
|  |  |  | G12C:IL-6 | G12C:Unstim | 2.697 | 1.98E-06 |
|  |  |  | G12C:DMOG | G12C:Unstim | 3.305 | 6.06E-09 |
| GO: 0046034 | ATP metabolic process | Culture | DMOG | IL-6 | 1.312 | 1.79E-07 |
|  |  |  | DMOG | Unstim | 0.790 | 5.78E-02 |
| GO: 1903578 | Regulation of ATP metabolic process | Culture | DMOG | IL-6 | 1.335 | 1.50E-02 |
|  |  |  | DMOG | Unstim | 1.831 | 4.84E-03 |

**Table S1.** List of context-specific significant pairwise comparison of the sum of LFQ intensity of proteins in the AP-MS from six GO terms of interest (see Fig. 5). For each GO term the sum of LFQ intensity was analyse with a three-way ANOVA followed by Tukey post-hoc tests (see shiny app). Only pairwise comparisons of ‘culture’ and ‘genetic’ context effects and their two-way interaction with a p-value inferior to 0.1 are displayed. For the Genetic:Culture contexts interaction, only comparison of groups with one term in common are displayed. The six GO terms were selected for comparison to phenotypic parameters related to proliferation, glucose metabolism and ATP metabolism.
